# Performance and oncologic safety of sentinel lymph node biopsy after neoadjuvant chemotherapy: Results from a tertiary care center in Lebanon

**DOI:** 10.1002/cam4.6868

**Published:** 2023-12-22

**Authors:** Mariam Zahwe, Aya El Sammak, Karim Ataya, Christelle Jabbour, Ayman Bsat, Bassel Hafez, Christine Atallah, Mira Kheil, Mohamad Ali Maktabi, Bashar Hassan, Vahe Panossian, Hazem Assi, Jaber Abbas, Eman Sbaity

**Affiliations:** ^1^ Faculty of Medicine American University of Beirut Beirut Lebanon; ^2^ Faculty of Health Sciences American University of Beirut Beirut Lebanon; ^3^ Department of Surgery American University of Beirut Medical Center Beirut Lebanon; ^4^ Faculty of Medicine University of Balamand Beirut Lebanon; ^5^ Department of Internal Medicine, Division of Hematology and Oncology Naef K. Basile Cancer Institute, American University of Beirut Medical Center Beirut Lebanon

**Keywords:** breast cancer, neoadjuvant therapy, sentinel lymph node biopsy

## Abstract

**Background:**

The feasibility of sentinel lymph node biopsy (SLNB) after neoadjuvant chemotherapy (NACT) in initially node‐positive patients is still controversial. We aim to evaluate the oncologic outcomes of SLNB after NACT and further compare the results between those who were initially node‐negative and node‐positive.

**Methods:**

This is a retrospective cohort that included patients diagnosed with invasive breast cancer and had surgical management between January 2010 and December 2016. Survival and recurrence data after 3–5 years were collected from patients' records. We divided patients into Group A who were initially node‐negative and had SLNB ± axillary lymph node dissection (ALND) and Group B who were node‐positive and had SLNB ± ALND.

**Results:**

Among initially node‐negative patients, 43 out of 63 patients did SLNB (Group A). However, among initially node‐positive patients only 28 out of 123 patients did SLNB (Group B). Out of the 71 patients who did SLNB after NACT, 26 patients had positive SLNs with only 14 patients who further underwent ALND. The identification rate of SLNB was 100% in Group A and 96.4% in Group B. The survival curves by nodal status showed no significant difference between overall survival and recurrence‐free survival at 5 years between patients in Group A versus Group.

**Conclusion:**

The results suggest that in properly selected patients, SLNB can be feasible after NACT. Our results resemble the reported literature on accuracy of SLNB after NACT and adds to the growing pool of data on this topic.

## 1 INTRODUCTION

The introduction of neoadjuvant chemotherapy (NACT) in the management of breast cancer has allowed for the downstaging of the tumor and thus de‐escalation of surgical treatment of the breast and axilla.^1^, ^2^, ^3^ Studies have shown that margin width had no impact on local recurrence and overall survival rates in patients that underwent NACT followed by breast conserving therapy.^4^ In addition, other potential benefits include assessing aggressiveness of the tumor by assessing degree of tumor response, reduce incidence of positive margins, and on some occasions decrease axillary dissections.^5^

Traditionally, axillary lymph node dissection (ALND) has been a mainstay of breast cancer therapy ever since the radical mastectomy was first described by William Halstead in 1907.^6^ This procedure, however, is associated with significant complications, owing to its radical nature. The most frequent complications include paresthesia due to transection of the intercostobrachial nerve, seroma formation, restricted range of motion of the arm, and lymphedema, occurring in 21.4% of women post‐ALND.^7^, ^8^, ^9^ However, as surgical interventions for breast cancer moved toward less invasive techniques, so has the surgical management of the axillary lymph nodes. ALND has been phasing out in favor of sentinel lymph node biopsy (SLNB) for breast cancer patients with clinically and radiologically negative lymph nodes^10^ and there is continuous interest in expanding the indications of SLNB in node‐positive patients, in an attempt to limit highly morbid ALNDs. The role of SLNB in patients with invasive breast cancer that have received NACT remains a controversial topic with highly variable and yet immature outcomes reported in the literature. A recent meta‐analysis reported that the FNR of SLNB in initially node‐positive patients who converted to negative post NACT is considered unacceptably high. However, authors have shown improved and acceptable FNR when specific selection criteria and techniques were applied.^11^

In particular, the utility of SLNB to confirm the absence of residual axillary nodal disease and thus avoid ALND in clinically node‐positive patients that turn node‐negative after receiving NACT is a major area of research and highly debatable. Many studies have demonstrated that as much as 40%–50% of clinically node‐positive patients can achieve complete histologic axillary response following NACT.^12^, ^13^ These studies support the potential to reduce the amount of unnecessary ALNDs and thus save breast cancer patients from their associated complications and morbidities. For optimal oncological results, the identification rate and false‐negative rates of SLNB after NACT should be >90% and <10%, respectively.^14^ A meta‐analysis by Cao et al published in 2021 reported that SLNB had an identification rate of 91% and a false‐negative rate of 15% in clinically node‐positive patients that were downstaged to node‐negative after receiving NACT,^11^ cautioning that while SLNB is a technically feasible method for confirming histologic nodal response after NACT, the false‐negative rate remains unacceptably high and thus sufficing with SLNB without completion of ALND needs to be better defined.

Hence, SLNB is a valid alternative to invasive ALND in a selected cohort of breast cancer patients who are initially node negative. In this retrospective single‐center study, we aim to compare the performance of SLNB after NACT in initially node‐negative versus node‐positive that transformed into a node‐negative axilla mainly in terms of the identification rate. We also aim to evaluate the oncologic outcomes of SLNB after NACT between the two groups.

## 2 METHODS

### 2.1 Study design and population

We conducted a retrospective cohort chart review of all patients diagnosed with breast cancer who underwent NACT and had surgical management at the American University of Beirut Medical Center (AUBMC) between January 2010 and December 2016. This study was approved by the Institutional Review Board of the medical center under the protocol number (BIO‐2019‐0391) with waiver of consent requirement. Inclusion criteria are as follows: (1) biopsy‐proven breast cancer patients (with or without lymph node involvement), (2) older than 18 years, treated at AUBMC between the defined study period, and (3) received NACT followed by SLNB. The exclusion criteria include: (1) no proof of histologic diagnosis by biopsy, and (2) did not receive any modality of the breast cancer treatment at AUBMC.

**FIGURE 1:**
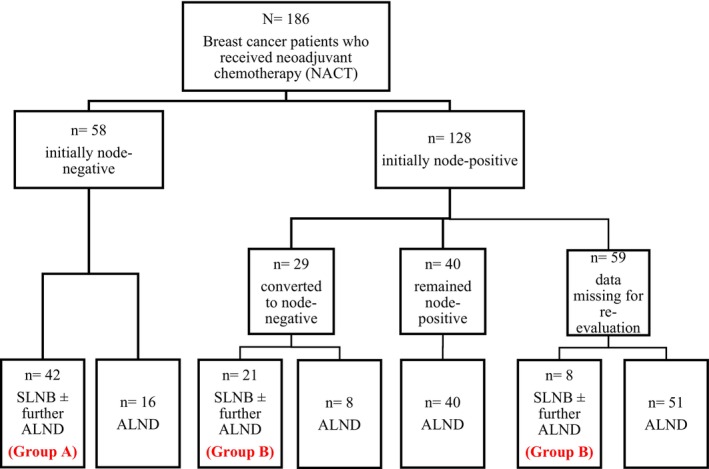
Algorithm of grouping patients who received neoadjuvant chemotherapy according to their nodal status. ALND, axillary lymph node dissection; NACT, neoadjuvant chemotherapy; SLNB, sentinel lymph node biopsy.

We determined the clinical nodal status of patients depending on ultrasound findings. If data on ultrasound were missing from the patient's medical records, we relied on the documented physical exam. We then classified patients into two groups based on the axillary involvement whether they further underwent ALND or not. Groups A for women with initially node‐negative breast cancer who received NACT followed by SLNB with or without ALND; and Group B for women with initially node‐positive breast cancer who converted to node‐negative after receiving NACT and underwent SLNB with or without ALND (Figure 1).

### 2.2 Treatment

Most patients received NACT with anthracycline or taxane‐based regimen. Following completion of NACT, surgeons repeated imaging evaluation for patients using mammogram, ultrasound, and MRI according to their decision. For those who were initially node‐positive and converted to negative nodal status on imaging, SLNB was offered for the majority of patients. The SLNB is done at AUBMC using two different approaches. The surgeon injects intraoperatively patent blue dye into the breast parenchyma. Another technique is injecting radioactive colloid material in the breast and using a gamma probe to detect and remove all radioactive axillary lymph nodes. In all cases of SLNB done after NACT at AUBMC, surgeons use the dual technique of radioactive colloid and blue dye according to the institutional policy. A skin incision is then done by the surgeon at the axilla. The surgeon then removes all radioactive and/or blue lymph nodes and submits to the pathology lab as sentinel lymph nodes (SLNs) for frozen section evaluation.

### 2.3 Outcomes

The aim of the study is to determine the identification rate of SLNB in lymph node involvement after NACT. The identification rate (%) was calculated as the number of patients with at least one identified SLN over the total number of patients who did SLNB  ×100%. We also aimed to report oncologic outcomes following these procedures including overall and disease‐free survival as well as nodal and distant recurrence at 5 years.

### 2.4 Data collection

Then data of the patient were collected from the electronic medical records of the patients by well‐trained research fellows and medical students. We used a uniform data collection sheet with multiple sections including demographics, age at diagnosis, tumor characteristics (pathological, clinical, and surgical stage), tumor size, ER status, PR status, HER‐2 receptor status, status of lymph node, chemotherapy received, radiotherapy, and surgical management. Data were extracted directly from the respective pathology, radiology, and laboratory reports. We collected oncologic data on overall survival and disease‐free survival outcomes as well nodal and distant recurrence. Data were audited to ensure integrity and accuracy of the data collection.

### 2.5 Statistical analysis

Analysis was done using SPSS v.23, and a *p* value of <0.05 was considered as statistically significant. We run frequencies for descriptive analysis. Categorical variables were reported as percentages and numerical variables as median with range. The chi‐squared test and Fisher exact test were used for univariate analysis of categorical variables. Bivariate analysis of the continuous variables was done using Student *t*‐test. Kaplan Meier method was used to analyze the outcomes of survival and recurrence at 5 years. The time to all oncologic events was calculated starting from date of surgery. We evaluated and compared the oncologic outcomes between two study groups using log‐rank test.

## 3 RESULTS

A total of 186 eligible breast cancer patients were included in our study. All the patients were women aged between 30 and 55 (mean age of 45.6) who received NACT. Only 71 (38.1%) of the patients underwent SLNB after NACT. Among these patients, 42 (59%) were initially node negative (Group A) and 29 (41%) were initially node‐positive patients (Group B). An algorithm of grouping patients was summarized in Figure 1.

The clinicopathological features of patients who underwent SLNB, and the treatment received are summarized in Table 1. The majority of patients in both Groups A and B had invasive ductal carcinoma with 83.3% and 93.1%, respectively. The majority of initially node‐positive patients were cN1 (62%). More than half of the patients in Groups A and B had breast cancer stage II. Patients in both Groups A and B received adjuvant radiotherapy and neoadjuvant Herceptin. Regarding the type of breast surgery done, more patients underwent partial mastectomy than total mastectomy in both Groups A and B.

**TABLE 1:** Clinicopathological features of patients who underwent SLNB, and the treatment received.

	Group A	Group B
Age at diagnosis (mean ± SD)	45.4 ± 11.749	45.8 ± 9609
Type of breast cancer (*n* = 71)		
Invasive ductal carcinoma	35 (83.3%)	27 (93.1%)
Invasive lobular carcinoma	3 (7.1%)	1 (3.4%)
Composite carcinoma	4 (9.4%)	1 (3.4%)
Mean tumor size (in cm)	3.1 ± 1.93	2.8 ± 1.12
Stage (*n* = 54)		
I	7 (21.9%)	3 (13.6%)
II	17 (53.1%)	13 (59.1%)
III	8 (25.0%)	6 (27.3%)
HER‐2 status (*n* = 71)		
Positive	13 (31.0%)	9 (31.0%)
Molecular subtype (*n* = 71)		
Luminal A	13 (31.0%)	8 (27.6%)
Luminal B	15 (35.7%)	17 (58.6%)
Her‐2 positive	7 (16.7%)	0 (0%)
Triple negative	7 (16.7%)	4 (13.8%)
Neoadjuvant herceptin (*n* = 71)		
Yes	14 (33.3%)	9 (31.0%)
Type of breast surgery (*n* = 71)		
Partial mastectomy	26 (61.9%)	18 (62.1%)
Total mastectomy	16 (38.1%)	11 (37.9%)
Adjuvant radiotherapy (*n* = 65)		
Yes	32 (82.1%)	23 (88.5%)

The technique used in SLNB varied between the use of radioactive colloid, blue dye or both (Table 2). In both Groups A and B, the majority of patients had their SLNB done using both techniques (75% in Group A and 52% in Group B), and no statistical difference was found between the two groups in terms of the SLNB technique used (*p* = 0.204).

**TABLE 2:** Technique and results of SLNB.

	Group A (*N* = 42)	Group B (*N* = 29)	*p*‐value
Technique of SLNB			
Radioactive colloid	5 (15.6%)	5 (23.8%)	0.204
Blue dye	3 (9.4%)	5 (23.8%)	
Both	24 (75%)	11 (52.4%)	
Number of patients with at least 1 SLN identified	42 (100%)	28 (96.6%)	0.231
Mean SLNs removed per patient	3.8 (±2.1)	4.3 (±2.2)	0.353
Number of patients with positive SLNs			
1 positive SLN	10	11	0.227
≥2 positive SLN	2	3	

The identification rate of SLNB in Group A who were initially node‐negative was 100%. For Group B who are initially node‐positive, the identification rate of SLNB was 96.6%. On average, participants in Group A had 3.8 identified SLN while participants in Group B had 4.3 identified SLN.

When looking at the number of patients with positive SLNs, 71.4% of patients in Group A had negative SLNs compared to 51.7% of patients in Group B. There was no statistical significance between both groups in the number of positive SLNs identified, with a p‐value of 0.227. In addition, only 11.1% of patients in Group A proceeded to ALND compared to 61.5% in Group B.

**FIGURE 2:**
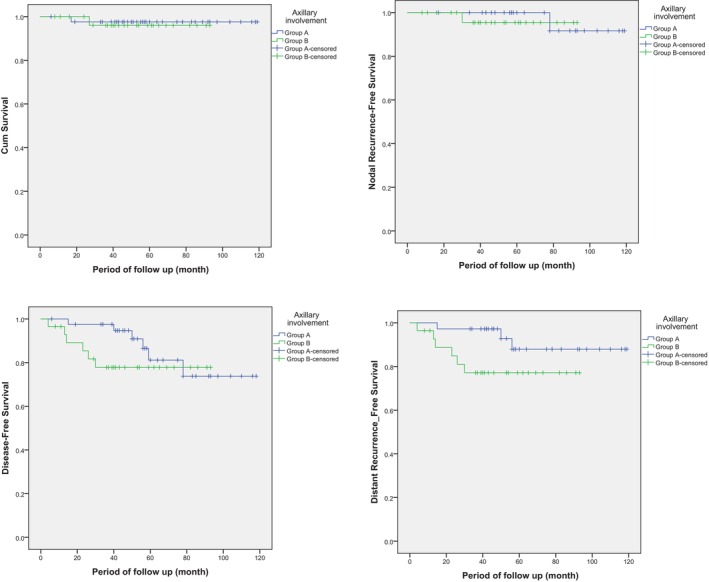
Survival curves for different oncologic outcomes in both Groups A and B.

The median follow‐up period was 53 months. Figure 2 displayed the survival curves for different oncologic outcomes in Groups A and B. There was one deceased case after 5 years in each group. The 5‐year‐overall survival of Group A was 97.6% compared to 81.2% in Group B. However, this difference was not statistically significant (*p* = 0.748). Disease‐free survival at 5 years was 81.2% in Group A compared to 77.8% in Group B. There was no nodal recurrence event at 5 years in Group A and only one case of nodal recurrence occurred in Group B. The 5‐year nodal recurrence free rate was 100% in Group A compared to 95.5% in Group B, with no statistical significance between the two groups (*p* = 0.725). At 5 years, distant recurrence occurred in three patients in Group A and six patients in Group B, resulting in 5‐year distant recurrence‐free survival rate of 87.9% in Group A and 77.1% in Group B (*p* = 0.089). Thus, there is no significant difference in the oncologic outcome at 5 years between patients who were initially node negative or those who were initially node‐positive and converted to node‐negative after NACT.

## 4 DISCUSSION

This study aimed at comparing the feasibility of oncologic outcomes of SLNB after NACT in patients who were initially node‐negative versus node‐positive after 5 years follow‐up. The results showed that SLNB can be feasible and safe to use in both group of patients after NACT.

All 42 of the clinically node‐negative patients in Group A had at least one identified SLN after NACT, attaining an identification rate of 100%. The identification rate is one of two key test parameters that quantify the reliability and accuracy of SLNB.^15^ Andreis et al. reported that SLNB accurately predicted the lymph node status of 101 clinically node‐negative patients out of 109, with an identification rate of 92.7%.^16^ Shirzadi and his colleagues assessed the reliability of SLNB after NACT in two subgroups: clinically node‐negative and clinically node‐positive patients. The pooled identification rate from 36 studies was 94% in the initially node‐negative subgroups. The study concluded that SLNB after NACT is rendered feasible and attains an adequate level of accuracy.^17^ Similarly, a systematic review evaluated the identification rate and false negative rates of SLNB after NACT and the pooled identification rate was 96%.^18^ A group of investigators studied the importance of the timing of SLNB before or after NACT and the identification rate of the latter was 95%.^19^ The study acknowledges the difference in identification rates between the two groups, but the latter is associated with a significantly less need of further axillary treatment, subsequently lowering the likelihood of associated consequences such as a 20% increase in morbidity.^16^ SLNB is a technique that could safely replace ALND as it is associated with minimal morbidity relative to ALND.^20^ According to Cody et al., in initially node‐negative patients who have had SLNB after NACT, the likelihood of axillary local recurrence after SLNB is <1%, thus ALND has no added advantages over SLNB when accounting for survival and/or morbidity.^15^, ^21^ This spares patients additional surgical operations and the possibility of further unnecessary complications.

Out of 29 patients in Group B, the number of patients with at least 1 SLN identified was 28 resulting in an identification rate of 96.6%. The success of SLNB depends strongly on the ability to identify the “true” SLNs, thus, identification rate is a crucial determining factor for SLNB technique. Our results were like the other studies which reported the identification rate in initially node‐positive patients who were converted to node‐negative after NACT as summarized by a recent meta‐analysis. The latter investigated the feasibility and reliability of SLNB in this sub‐population and reported a pooled estimate of identification rate of 91% where it fluctuated from 72% to 100% in the included studies.^11^ This high identification rate that we reported might be associated with the fact that majority of our patients had SLNB using dual technique (52.4%). The American College of Surgeons Oncology Group (ACOSOG) Z1071 trial was one the initial trial to introduce the efficacy of SLNB after NACT, where the SLN mapping with both blue and radiolabeled colloid mapping agents was recommended to maximize the likelihood of SLN.^12^ According to Boughey et al, the type of mapping agent used in SLNB was the only factor associated with the failure to identify a SLN where using a dual agent mapping showed a higher identification rate compared to single‐agent mapping (93.8% vs. 88.9%, *p* = 0.048).^22^

During a median follow‐up of 53 months, our results showed that there was not a significant difference while comparing the nodal recurrence‐free and distant recurrence‐free survival rates between the two groups. These findings add to the existing literature showing that the clinical outcomes of clinically node‐negative or node‐positive patients do not largely vary after undergoing SLNB only. According to a study by Barrio et al. there was only one event of nodal recurrence (0.4%) in the 205 initially node‐positive patients who underwent adjuvant radiotherapy and omitted ALND.^23^ The GANEA‐2 trial by Classe et al. displayed similar results where only 1 out of 419 clinically node‐negative patients reported an axillary recurrence after a 3‐year follow‐up post‐SLNB after NACT.^24^ Of note, Piltin et al. showed that the regional recurrence of the SLNB‐only patients was 99.1% while that of the ALND patients was 96.4%, despite the lack of statistical significance. However, the recurrence‐free survival rates for the SLNB‐only patients were significantly better than that of the patients who underwent ALND (*p* = 0.003), an operation known to implicate higher rates of morbidity.^25^ These studies provide preliminary evidence that SLNB after NACT, regardless of the clinical node status of patients, is effective and safe without any further need for ALND.

Recently, Sanchez et al. analyzed the oncological outcomes of 399 patients according to their SLNB status. During a 3‐year median follow‐up, the overall survival, distant disease‐free survival, and regional disease‐free survival rates were 95.5%, 92.2%, and 94.2% in initially node‐negative patients while that of the initially node‐positive patients were 93%, 84.8%, and 87.9%, respectively.^26^ Our data revealed similar results displaying a lack of significant difference between the two groups. Our results are in line with a study by Damin et al. that showed lack of significance between oncological outcomes, indicating that ALND can be omitted.^27^ Our observations are further reinforced by Jun Choi et al., who conducted a detailed analysis between initially node‐negative patients who underwent SLNB only, patients with a negative SLN status who underwent ALND, and patients with no residual axillary metastasis who underwent ALND. The study emphasizes that ALND has no additional oncological advantages over SLNB and allows for the reduction of axillary morbidity rendering SLNB as not only feasible but can also safely replace ALND.^28^

## 5 CONCLUSION

The results suggest that in properly selected patients, SLNB can be feasible after NACT. Our results resemble the reported literature on feasibility of SLNB after NACT and add to the growing pool of data on this topic. However, further study with a larger sample size in a prospective manner is needed to have more solid and generalizable conclusion, and to test for the safety of SLNB after NACT.

### 5.1 Strengths and limitations

This study aimed to fill a gap in the literature where there are limited data on the performance of SLNB after NACT in initially node‐positive patients who converted to node‐negative in Lebanon. We provided a long‐term oncologic outcome among a cohort of patients who received NACT and then did SLNB. However, this present study has limitations because of its retrospective design and the relatively small sample size making it difficult to reach a conclusion that is generalized. Although AUBMC is a tertiary referral center for breast cancer, data collected from one site are another limitation to this study. In addition, there might be a selection bias because it was not clear how the surgeons were deciding which patients undergo SLNB, ALND, or both. Moreover, as not all patients underwent ALND we were not able to compute the false‐negative rate of SLNB.

## AUTHOR CONTRIBUTIONS

**Mariam Zahwe:** Methodology (lead); project administration (lead); software (lead); supervision (lead); writing – original draft (lead); writing – review and editing (lead). **Aya El Sammak:** Data curation (equal); visualization (equal); writing – original draft (equal). **Karim Ataya:** Data curation (equal); visualization (equal); writing – original draft (equal). **Christelle Jabbour:** Data curation (equal); visualization (equal); writing – original draft (equal). **Ayman Bsat:** Data curation (equal); formal analysis (supporting); visualization (equal); writing – original draft (equal). **Bassel Hafez:** Data curation (equal); resources (equal); writing – original draft (equal). **Christine Atallah:** Data curation (equal); writing – original draft (equal). **Mira Kheil:** Data curation (equal); writing – original draft (equal). **Mohamad Ali Maktabi:** Data curation (equal); writing – original draft (equal). **Bashar Hassan:** Data curation (equal); writing – original draft (equal). **Vahe Panossian:** Data curation (equal); writing – original draft (equal). **Hazem I. Assi:** Resources (equal); writing – review and editing (equal). **Jaber Abbas:** Resources (equal); writing – review and editing (equal). **Eman Sbaity:** Conceptualization (lead); data curation (lead); resources (lead); supervision (lead); writing – review and editing (lead).

## CONFLICT OF INTEREST STATEMENT

The authors declare no conflicts of interest.

## ETHICS STATEMENT

This study was approved by the institutional review board at the American University of Beirut (IRB number: BIO‐2019‐0391).

## PATIENT CONSENT STATEMENT

The IRB waived patient consenting for this study because of its retrospective design on condition that the data is de‐identified to secure confidentiality of data.

## Data Availability

The datasets analyzed in the current study are available on request from the corresponding author.
